# Supplementary material for: A Comprehensive Assessment of Ultraviolet-Radiation-Induced Mutations in Flammulina filiformis Using Whole-Genome Resequencing
Source: J Fungi (Basel). 2024 Mar 20;10(3):228. doi: 10.3390/jof10030228 (PMC10971301; doi:10.3390/jof10030228)
Supplement: Supplementary file 1 [file jof-10-00228-s001.zip › Supplementary Material S8/KEGG annotation/out/64381550635650.os/KO/out_map/map04261.html]

KEGG PATHWAY: Adrenergic signaling in cardiomyocytes - Reference pathway


|  |  |
| --- | --- |
| **Adrenergic signaling in cardiomyocytes - Reference pathway** |  |

[
Pathway menu
| Organism menu
| Pathway entry
| Show description
| User data mapping
]

Cardiac myocytes express at least six subtypes of adrenergic receptor (AR) which include three subtypes of beta-AR (beta-1, beta-2, beta-3) and three subtypes of the alpha-1-AR (alpha-1A, alpha-1B, and alpha-1C). In the human heart the beta-1-AR is the pre- dominate receptor. Acute sympathetic stimulation of cardiac beta-1-ARs induces positive inotropic and chronotropic effects, the most effective mechanism to acutely increase output of the heart, by coupling to Gs, formation of cAMP by adenylyl cyclase (AC), and PKA- dependent phosphorylation of various target proteins (e.g., ryanodine receptor [RyR]; phospholamban [PLB], troponin I [TnI], and the L-type Ca2+ channel [LTCC]). Chronic beta-1-AR stimulation is detrimental and induces cardiomyocyte hypertrophy and apoptosis. beta-2-AR coupled to Gs exerts a proapoptotic action as well as beta-1-AR, while beta-2-AR coupled to Gi exerts an antiapoptotic action.

|  |  |
| --- | --- |
| Reference pathway | 100% |
